# A Unique Mouse Model of Early Life Exercise Enables Hippocampal Memory and Synaptic Plasticity

**DOI:** 10.1038/s41598-020-66116-4

**Published:** 2020-06-08

**Authors:** Autumn S. Ivy, Tim Yu, Enikö Kramár, Sonia Parievsky, Fred Sohn, Thao Vu

**Affiliations:** 1 Department of Pediatrics, University of California-Irvine School of Medicine, Irvine, USA; 2 School of Biological Sciences, University of California-Irvine, Irvine, USA; 3 Department of Neurobiology and Behavior, University of California-Irvine, Irvine, USA; 4 Center for the Neurobiology of Learning and Memory, Irvine, CA USA

**Keywords:** Hippocampus, Long-term memory, Spatial memory, Long-term potentiation

## Abstract

Physical exercise is a powerful modulator of learning and memory. Mechanisms underlying the cognitive benefits of exercise are well documented in adult rodents. Exercise studies targeting postnatal periods of hippocampal maturation (specifically targeting periods of synaptic reorganization and plasticity) are lacking. We characterize a model of early-life exercise (ELE) in male and female mice designed with the goal of identifying critical periods by which exercise may have a lasting impact on hippocampal memory and synaptic plasticity. Mice freely accessed a running wheel during three postnatal periods: the 4^th^ postnatal week (juvenile ELE, P21–27), 6^th^ postnatal week (adolescent ELE, P35–41), or 4^th^-6^th^ postnatal weeks (juvenile-adolescent ELE, P21–41). All exercise groups increased their running distances during ELE. When exposed to a subthreshold learning stimulus, juv ELE and juv-adol ELE formed lasting long-term memory for an object location memory task, whereas sedentary and adol ELE mice did not. Electrophysiological experiments revealed enhanced long-term potentiation in hippocampal CA1 in the juvenile-adolescent ELE group. I/O curves were also significantly modulated in all mice that underwent ELE. Our results suggest that early-life exercise, specifically during the 4^th^ postnatal week, can enable hippocampal memory, synaptic plasticity, and alter hippocampal excitability when occurring during postnatal periods of hippocampal maturation.

## Introduction

Exercise during adulthood is highly effective in improving cognitive functions in both humans and rodents^1,2^. In children, clinical studies positively associate higher physical activity levels with improved working memory^3^, academic performance^4^, and structural brain health^5^. Preclinical exercise models using adult rodents have identified underlying neurobiological mechanisms by which exercise may improve hippocampus-dependent memory: exercise augments neurogenesis^6^, vascularization^7^, synapse number^8^, facilitates long-term potentiation (LTP) in DG^9–11^, (but see^12^ regarding female mice) and in certain cases, area CA1^13,14^ (but see^15^), and induces a number of synaptic genes and proteins, including the key plasticity-modulating growth factor brain-derived neurotrophic factor (BDNF;^16,17^). There is some evidence that exercise may engage similar plasticity mechanisms in the developing brain^18^, however, studies focusing on the neurobiological sequelae of early-life exercise are lacking. Yet, it is precisely during early-life development periods that the effects of exercise may have the most long-term benefit on memory and cognitive ability throughout the lifespan^19^.

The cognitive and neurotrophic effects of exercise during adulthood are transient, seeming to fade in days to weeks. For example, one study exercised adult rats for three weeks then performed cognitive testing two-weeks after exercise cessation. These rats had weaker memory performance on a radial arm maze task after being sedentary for two weeks post-exercise. Coupled with this finding were gradual reductions in BDNF mRNA and protein in hippocampus, eventually returning to baseline levels 14 days post-exercise cessation^20^. In contrast, more recent studies focused on early-life exercise find that benefits to spatial memory and hippocampal plasticity persist into adulthood when the exercise is initiated in adolescence^21–23^. This suggests an enduring effect of the adolescent exercise experience on learning and memory^24^. Exact temporal windows by which an early life exercise experience can promote enduring changes in neuronal function, hippocampal plasticity, and behavior have not yet been thoroughly investigated.

The mammalian brain undergoes protracted postnatal development in a region- and functional network-specific manner^25,26^. In humans, neuronal differentiation and synaptogenesis within hippocampus reach adult levels around 3–5 years of age^27^, which is around the time children are able to reliably form long-term spatial memory^28^. In rodents, the hippocampus reaches milestones in synapse density and circuit refinement during the 3^rd^ to 4^th^ postnatal weeks (reviewed in^29,30^) which coincides with development of stable long-term potentiation (LTP^31,32^) and establishment of learning and memory functions^33,34^. A characteristic of postnatal brain maturation is synaptic overproduction and subsequent pruning^35^, thus rendering these developmental processes exquisitely sensitive to environmental experiences^36^. Normal processes of postnatal neurodevelopment are likely guided by specific activity- and experience-dependent patterns of gene expression that ultimately inform cell function within circuits^37,38^. It is thus reasonable to target defined periods of hippocampal structural and functional maturation, using biological mouse models, to uncover temporal windows of postnatal development by which hippocampal function can be persistently modulated by the experience of exercise.

In this study we developed a model of voluntary physical activity during specific early life developmental periods in mice (early-life exercise, or ELE) to address the hypothesis that the timing of exercise during postnatal hippocampal maturation can lead to enduring benefits in cognitive function and synaptic plasticity. We will refer to the 4^th^ week of life in a mouse (postnatal days 21–27) as a “juvenile” period, distinct from adolescence (5^th^-6^th^ postnatal weeks, as reviewed in^39,40^). We show that both male and female mice undergoing exercise during the juvenile period exhibit enabled hippocampus-dependent spatial memory, increased long-term potentiation and changes in synaptic excitability. Importantly, our ELE model can be used to uncover temporally specific molecular mechanisms engaged by ELE to influence neuronal function and behavior in a lasting manner.

## Results

### Running behavior and weight gain characteristics of the early-life exercise (ELE) mouse model

**Figure 1:**
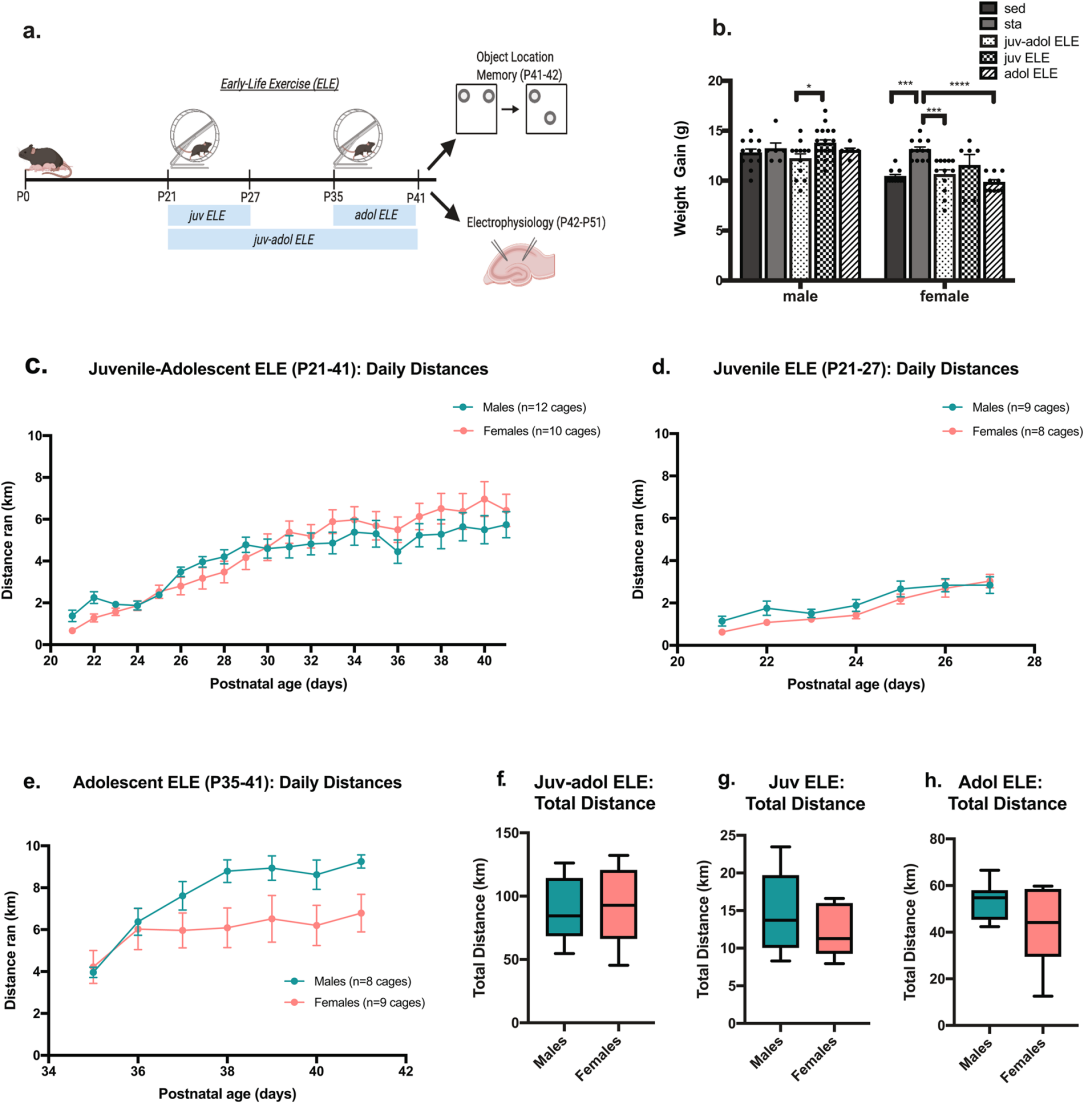
Experimental Design, Running Behavior and Weight Trends in Early Exercise Model. (**a**) Experimental design diagram. Upon weaning on P21, wild-type male and female mice in the juv ELE and juv-adol ELE were housed in cages equipped with voluntary running wheels for 1 week (juv ELE) or 3 weeks (juv-adol ELE). Adol ELE mice were housed in cages on P35 and allowed to run freely for 1 week. All mice were then tested in object location memory (OLM) or sacrificed for electrophysiology studies. (**b**) A significant difference in weight gained during ELE periods was observed between male juv ELE and juv-adol ELE groups. Female stationary mice gained significantly more weight than sedentary, juv-adol ELE, and adol ELE mice. (**c**–**e**) All ELE mice significantly increased their running distance throughout the early-life period of running wheel access. (**f**–**h**) No sex differences in total cumulative distance ran in any ELE group. Data were analyzed using one-way ANOVA with *post-hoc* comparisons or Student’s t tests: **p ≤ 0.01, and ***p ≤ 0.001. n = 8–12 exercise cages per group, 2 mice per cage. Experimental design diagram generated with Biorender.com academic subscription.

Given the developmental timing of voluntary exercise in this model, we postulated that there would be important sex-specific and running group-specific differences in weight gain and distance ran across time. These differences would depend upon the *timing* (juvenile and/or adolescence) and *duration* (1- or 3-weeks) of the early-life exercise exposure. Male and female mice were either weaned directly into cages without a running wheel (sed), equipped with a running wheel on P21 (juv ELE and juv-adol ELE groups), or placed into cages without running wheels until P35 (adol ELE group, Fig. 1a). A separate group of mice were housed with a stationary wheel during P21–41 (sta). ELE mice were allowed to run for either one week (juv ELE and adol ELE groups) or three weeks (juv-adol ELE group, Fig. 1a). All mice were pair-housed to avoid the effects of social isolation stress^41^. Mice tended to run during the dark phase. For all running groups, the exercise exposure took place either just before or during adolescent growth spurts in mice (usually occurring during the 4^th^-5^th^ postnatal weeks), thus weight gained between P21 and P42 was compared across sed and ELE groups and between sexes. Two-way ANOVA revealed a significant interaction between sex and ELE group, suggesting that ELE has differential effects on weight gain dependent upon sex (Fig. 1b; *F*_*(4,94)*_ = 2.48, *p* = 0.049). *Post hoc* multiple comparisons revealed that in males, juv-adol ELE mice had significantly less weight gain than juv ELE mice (*p* = 0.045). Female mice housed with a stationary wheel (sta) had significantly greater weight gain than sed (*p* = 0.0005), juv-adol ELE (*p* = 0.0007), and adol ELE female mice (*p* < 0.0001). These findings suggest that a longer duration of ELE exposure (three weeks) during juvenile/adolescence significantly reduces weight gain in male mice, whereas in female mice, presence of a stationary wheel led to greater weight gain than mice in sedentary cages without running wheel and mice that ran during adolescence.

We next addressed the question whether there are sex differences in daily and cumulative running distances in our three ELE groups. Juv-adol ELE male and female mice gradually increased their daily running distance over the 3-week period (Fig. 1c). A two-way repeated-measures ANOVA revealed a significant effect of postnatal day (*F*_*(3,57)*_ = 41.95, *p* < 0.0001) and significant interaction with sex (*F*_*(20,400)*_ = 2.15, *p* = 0.003). There was no independent effect of sex on daily running distance (*F*_*(1,20)*_ = 0.15, *p* = 0.70), nor was there a significant difference between sexes in total cumulative distance ran (*p* = 0.70). In evaluating the exercise behavior of mice running for the shorter duration (1-week), we found that postnatal day had a significant effect on daily running distance in juv ELE mice (Fig. 1d; *F*_*(3,51)*_ = 35.93, *p* < 0.0001). There was no independent effect of sex on running distance (*F*_*(1,15)*_ = 1.11, *p* = 0.31) and no interaction (*F*_*(6,90)*_ = 1.20, p = 0.31). In contrast, adol ELE mice had a statistically significant interaction of postnatal day x sex (Fig. 1e; *F*_*(6,84)*_ = 4.77, *p* = 0.0003). Although female adol ELE mice trended toward less running toward the end of the adolescent running period this did not reach significance (*p* > 0.05). Similar to all other running groups, adol ELE mice also significantly increased their running across postnatal days (*F*_*(4,58)*_ = 24.87, *p* < 0.0001). We next compared daily and cumulative running distances in 1-week runners, expecting that running distance would be significantly greater in adol ELE mice when compared to juv ELE mice (regardless of sex) given their developmental stage and body mass differences when entering running cages. There were no sex differences in total running distance within juv and adol ELE groups (Fig. 1f–h); however, cumulative distance traveled over their respective running periods was significantly greater in the adol ELE group when compared to the juv ELE group, and this was true for both sexes (males: *t*_(15)_ = 11.96, *p* < 0.0001; females: *t*_(14)_ = 4.69, *p* = 0.0004). Total distances ran per cage over the duration of the exercise exposures were the following: juv-adol ELE mice; males = 87.8 ± 6.9 km, females = 92.2 ± 9.3 km; juv ELE mice; males = 14.7 ± 1.8 km, females = 12.29 ± 1.2 km; adol ELE mice; males = 53.6 ± 2.8 m, females = 41.8 ± 6.2 km. In sum, all running groups and both sexes of mice gradually and significantly increased their running distances across time. The cumulative amount of voluntary running throughout the running period was dependent on when early life exercise was initiated (in the juvenile period vs in adolescence).

### **Juvenile exercise enables long-term memory in both male and female mice in a lasting manner**

Previous studies have demonstrated that in adult mice, a minimum of 2–3 weeks of exercise is required for exercise-induced improvements in long-term memory^42,43^; however, a minimum amount of exercise needed for benefits to learning and memory in juvenile and adolescent mice has not been established. Furthermore, in adult mice, the exercise-induced memory benefits start to fade by 3 days after exercise ceases^43^. Whether ELE benefits hippocampal memory for a longer (or shorter) duration than in adult mice after the cessation of exercise is an unanswered question. We therefore tested the following hypotheses: 1) that a learning stimulus typically insufficient for long-term memory formation in sedentary wild-type mice can become sufficient after ELE (as it does after 2–3 weeks of exercise in adulthood), and 2) the early-life timing of exercise during juvenile and/or adolescent periods will have a lasting impact on the duration of ELE-induced improvements in long-term memory.

**Figure 2:**
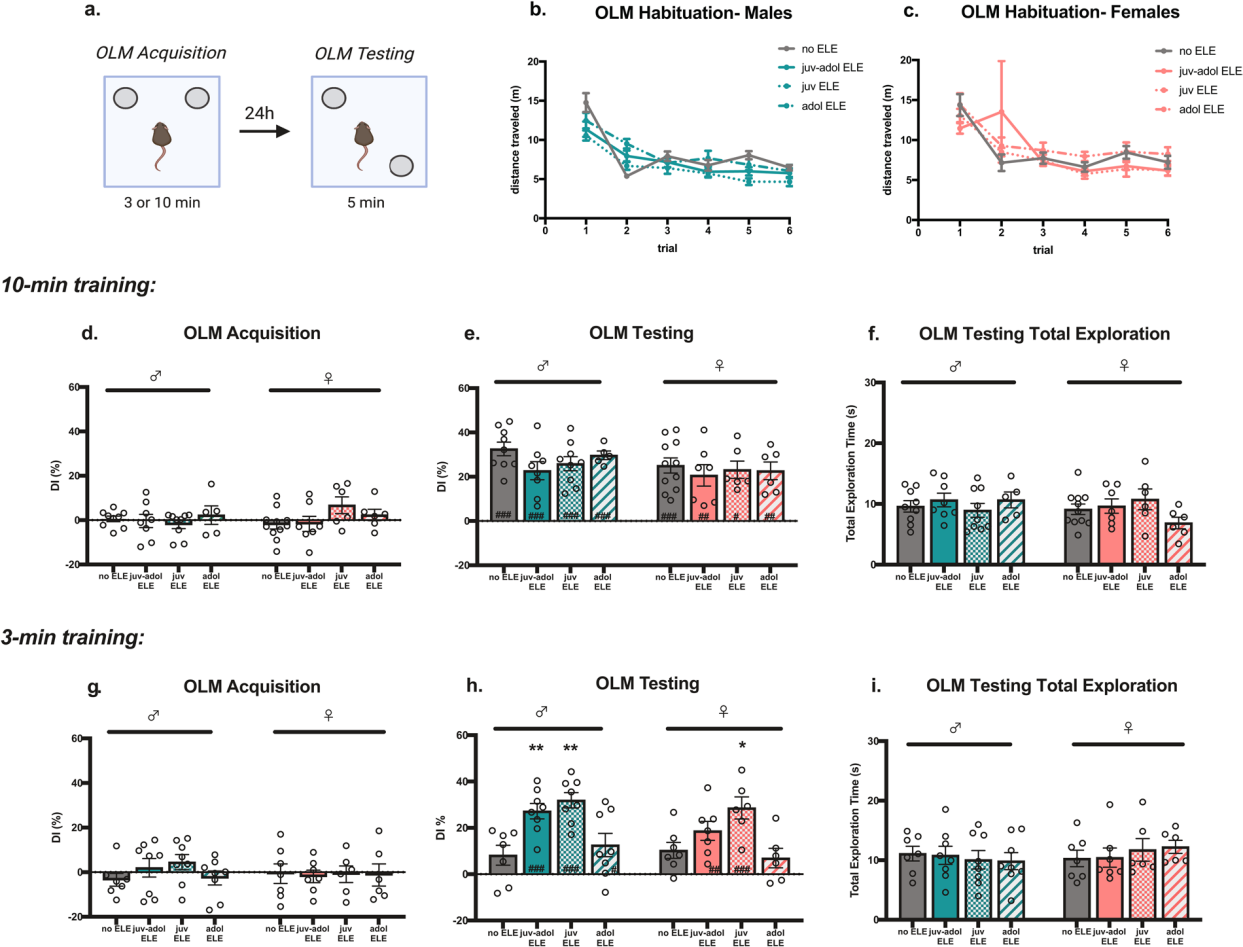
Juvenile-adolescent ELE results in improved novel object recognition memory in male and female mice. (**a**) OLM experimental design diagram. (**b,c**) Habituation analysis revealed male and female adolescent mice similarly habituated to OLM chambers, regardless of group, as demonstrated by significantly reduced distance traveled across trials. (**d**) OLM acquisition in 10-min trained male and female mice demonstrated no significant object discrimination. (**e**) During OLM testing, all mice demonstrated significant exploration of the novel-placed object, and there were no significant differences in DIs between groups. (**f**) No differences in total exploration during OLM testing in 10-min trained mice. (**g**) Mice trained for 3-min during OLM acquisition did not demonstrate object preference. (**h**) Juv ELE and juv-adol ELE male mice, and juv ELE female mice,  had significant preference for the object placed in a novel location when compared to sedentary controls, whereas adol ELE mice did not. (**i**) Total exploration times during OLM testing in 3-min trained mice did not differ across groups or sexes. P*ost hoc* comparisons vs ‘no ELE’ group: (*p < 0.05; **p < 0.01; ***p < 0.005 vs no ELE) and within group comparisons vs OLM training DI: (^#^p < 0.05; ^##^p < 0.01; ^###^p < 0.005 vs training DI). N = 6–12 mice per group. Experimental design diagram generated with Biorender.com academic subscription.

Hippocampus-dependent learning and memory was assessed using an Object Location Memory (OLM) task, a non-aversive spatial memory task that relies on the rodent’s innate preference for exploring novel placement of objects^44^. All mice were tested in OLM during adolescence (P41–42). Sedentary and stationary wheel-housed mice had no significant differences in their OLM performances, so these groups were combined (no ELE). Mice were exposed to two identical objects for either 3-min or 10-min during the OLM acquisition phase, followed by a 5-min OLM retention test performed 24 hours later (Fig. 2a). During OLM testing one of the objects was placed in a novel location and times spent exploring familiar and novel object locations were quantified and expressed as a discrimination index (DI). All mice were habituated to the OLM chambers for three consecutive days, twice per day, and demonstrated habituation by significantly reducing distance traveled across trials regardless of exercise group (Fig. 2b,c; males: main effect of habituation trial: *F*_(3.6, 146.2)_ = 75.76, *p* < 0.0001; no effect of exercise group: *F*_(7, 83)_ = 1.48, *p* = 0.19; group x trial interaction: *F*_(15,203)_ = 2.63, *p* = 0.001. females: main effect of habituation trial: *F*_(1.201, 45.38)_ = 8.93, *p* = 0.003; no effect of exercise group: *F*_(3,40)_ = 0.55, *p* = 0.65; no group x trial interaction: *F*_(15,189)_ = 0.81, *p* = 0.67).

In the sub-threshold OLM design used for this study, the 3-min training exposure (acquisition phase) is a duration previously established as sub-threshold for supporting long-term memory formation in adult mice, whereas the 10-min training exposure is typically sufficient^45^. Our first experiments tested whether 10-min of training during OLM acquisition was also sufficient for long-term memory formation during adolescence. No object preference was observed in any of the groups, as indicated by average DIs close to 0 (Fig. 2d; males: *F*_(3, 27)_ = 0.47, *p* = 0.70; females: *F*_(3,27)_ = 1.93, *p* = 0.15). During OLM testing 24 h later, all 10-min trained groups exhibited strong preference for novel-placed objects, as demonstrated by a significant increase in testing DI when compared to acquisition DIs (Fig. 2e; males: main effect of OLM session: *F*_(1,27)_ = 143.5, *p* < 0.0001; no effect of running group: *F*_(3,27)_ = 2.06, *p* = 0.12; and no interaction: *F*_(3,27)_ = 0.73, *p* = 0.54. females: main effect of OLM session: *F*_(1,26)_ = 66.4, *p* < 0.0001; no effect of running group: *F*_(3,26)_ = 0.78, *p* = 0.52; and no interaction: *F*_(3,26)_ = 0.80, *p* = 0.50). Running exposure did not significantly improve memory performance during OLM testing, suggesting a possible plateau of learning reached by all groups after being trained for 10-min the day prior (males: *F*_(3,27)_ = 1.71, *p* = 0.19; females: *F*_(3,26)_ = 0.23, *p* = 0.87). Further, there were no differences in total object exploration times during OLM testing (Fig. 2f; males: *F*_(3,27)_ = 0.56, *p* = 0.65; females: *F*_(3,26)_ = 1.65, *p* = 0.20). These data suggest that performance in the OLM task can be used as a robust measure of long-term memory formation in adolescent male and female mice.

3-min of OLM training has been demonstrated to be insufficient for long-term memory formation in non-exercised adults but can become sufficient if immediately preceded by exercise^42,43^. Given the exercise-induced improvements in long-term spatial memory in adult mice, we sought to determine whether this phenomenon also holds true during adolescence. All groups trained for 3-min did not exhibit significant object preference during OLM acquisition (Fig. 2g, males: *F*_(3, 27)_ = 1.17, *p* = 0.34; females: *F*_(3,24)_ = 0.03, *p* = 0.99). Sedentary male and female mice had significantly lower DIs than 10-min trained sedentary mice, suggesting that 3-min trained, non-exercised adolescent (P42) mice do not form strong long-term memory for object location (unpaired *t*-tests, males: t_(14)_ = 4.73, *p* = 0.0003; females: t_(16)_ = 2.90, *p* = 0.01). Comparisons of corresponding OLM testing DI to acquisition DI revealed that any exposure to early-life exercise in male mice leads to significant novel object location preference during testing, whereas in female mice, only the juv ELE and juv-adol ELE groups had significant object preference during testing when compared to training (Fig. 2h; males: no ELE: *p* = 0.18; juv-adol ELE: *p* = 0.0002; juv ELE: *p* < 0.0001; adol ELE: *p* = 0.03; females: no ELE: *p* = 0.15; juv-adol ELE: *p* = 0.002; juv ELE: *p* < 0.0001; adol ELE: *p* = 0.47). When comparing each exercise group to sedentary controls, male mice that underwent exercise during the juvenile period (juv ELE and juv-adol ELE) showed strong preference for novel object location when trained for 3-min (Fig. 2h; *F*_(3, 27)_ = 7.78, *p* = 0.0007; no ELE vs juv-adol ELE: *p* = 0.009; no ELE vs juv ELE: *p* = 0.001) whereas adol ELE mice did not (no ELE vs adol ELE: *p* = 0.84). In females, only the juv ELE group had significantly greater preference for novel object location when compared to sedentary controls (Fig. 2h; *F*_(3,22)_ = 5.23, *p* = 0.007; no ELE vs juv ELE: *p* = 0.01; no ELE vs juv-adol ELE: *p* = 0.38; no ELE vs adol ELE: *p* = 0.92). All groups of 3-min trained mice spent similar times exploring objects during OLM testing (Fig. 2i; males: *F*_(3,27)_ = 0.16, *p* = 0.92; females: *F*_(3,22)_ = 0.39, *p* = 0.76).

Finally, a three-way ANOVA was performed to determine the impact of and interactions between all nominal variables on the DIs of each group, including sex as a biological variable (total of three nominal variables: OLM training duration, ELE group, and sex; measurable variable: DI). Each individual variable was a significant source of variation (OLM training duration: *F*_(1,102)_ = 13.23, *p* = 0.0004; sex: *F*_(1,102)_ = 4.74, *p* = 0.03; ELE group: *F*_(3,102)_ = 4.42, *p* = 0.006). There was a significant interaction between training duration and ELE group, supported by prior analyses (*p* < 0.0001); however, there were no other significant interactions (ELE group x sex, training duration x sex, ELE group x training duration x sex: all with *p* > 0.05). These results suggest that in both sexes, exercise taking place during a specific juvenile developmental period (P21–27) is sufficient for enhancing long-term memory for an OLM acquisition trial that is usually insufficient for long-term memory in sedentary controls, and furthermore, juv ELE-induced enabling of long-term memory is present at least 2 weeks after the juvenile exercise period ends.

### **Synaptic plasticity and hippocampal excitability in CA1 are modulated in male mice after early-life exercise**

**Figure 3:**
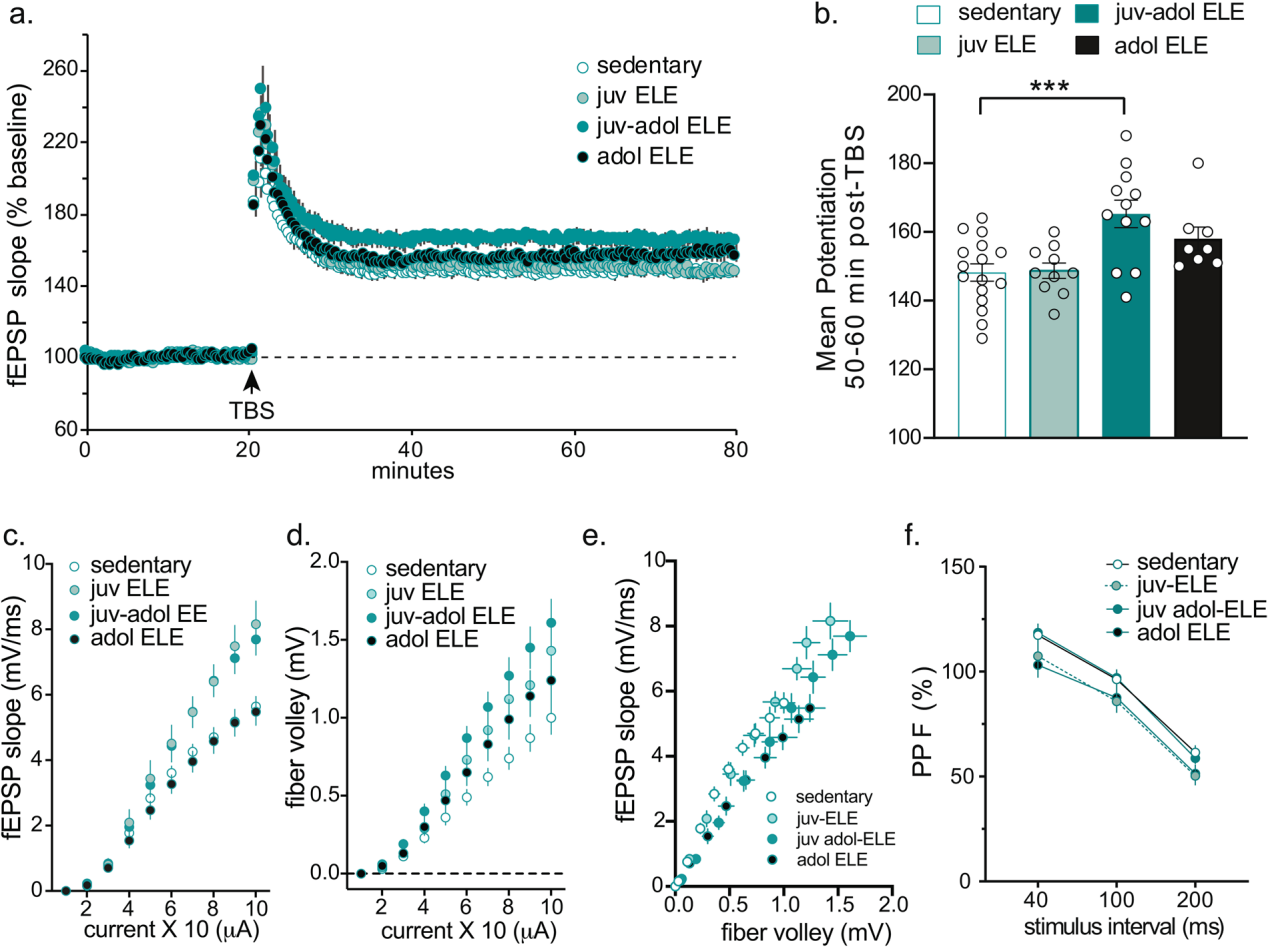
Early-life exercise enhances hippocampal synaptic plasticity in CA1. (**a**) Hippocampal slices from mice undergoing 3 weeks of exercise (juv-adol ELE) had a significant increase in LTP in response to theta burst stimulation (TBS) compared to sedentary controls, 1-week exercise in juvenile mice during P21–27 (juv ELE) and 1-week exercise in adolescent mice during P35–41 (adol ELE). (**b**) This enhanced potentiation in juv-adol ELE slices was sustained 50–60 min post-TBS. (**c**) I/O curve plotting EPSP slope against current generated in the Schaffer collaterals showed a significant increase in fEPSP slopes in both juv ELE and juv-adol ELE compared to adol ELE and sedentary controls. (**d**) I/O curve plotting EPSP slope against presynaptic fiber volleys also shows significant effect of early-life exercise on fiber volley amplitude compared to sedentary controls. (**e**) I/O curve plotting the relationship between fiber volley amplitude and EPSP slope. (**f**) No significant differences were found between groups following paired-pulse facilitation. ***p ≤ 0.005.

Prior studies have confirmed that memory for object location requires an intact, functioning dorsal hippocampus^46^. Therefore, synaptic plasticity was examined in the CA3-CA1 Schaffer collateral pathway of the hippocampus in sedentary and ELE mice, to test the hypothesis that ELE also leads to an enhancement in synaptic strength in the same region involved in enabled memory performance after juv and juv-adol ELE. Long-term potentiation (LTP) studies were performed in acute hippocampal slices from juvenile, adolescent, and juvenile-adolescent ELE mice. Only male mice were used in electrophysiological studies. Mice were sacrificed between P42-P51. LTP was induced with a single train of 5 theta bursts to Schaffer collateral inputs and extracellular field excitatory post-synaptic potentials (fEPSPs) were recorded from apical dendrites of CA1b. LTP was notably increased in juv-adol ELE mice but not in the juv ELE or adol ELE groups (Fig. 3a; *F*_(3,42)_ = 7.20, *p* = 0.0005). The enhanced LTP in the juv-adol ELE group was sustained for 50–60 minutes post-TBS (Fig. 3b; compared to sedentary: *p* = 0.0006). This finding is consistent with prior studies demonstrating that one-week of voluntary physical activity is typically an insufficient duration of exercise for increasing LTP in adult rodents (specifically in dentate gyrus^10^).

Input/output curves were generated by recording fEPSP slopes in response to increasing stimulus intensities in order to test the hypothesis that ELE may enhance hippocampal synaptic transmission. The input/output relationship for fEPSP versus stimulus intensity (current) revealed a main effect of ELE group (Fig. 3c; *F*_(3,38)_ = 5.93, *p* = 0.002), effect of stimulus intensity (*F*_(9,342)_ = 444.3, *p* < 0.0001), and significant interaction (*F*_(27,342)_ = 5.08, *p* < 0.0001). *Post-hoc* analysis revealed enhanced fEPSP slopes in the juv ELE and juv-adol ELE groups compared to controls, seen only at the higher stimulus intensities (between 7–10 (x10) uA; *p* < 0.05). Additionally, afferent fiber volley amplitudes (which are considered to represent presynaptic physiological responses within the slice) were also plotted against stimulus intensity, and again demonstrated main effects of ELE group (Fig. 3d; *F*_(3,420)_ = 34.07, *p* < 0.0001), main effect of stimulus intensity (*F*_(9,420)_ = 148.2, *p* < 0.0001), and significant interaction (*F*_(27,420)_ = 2.02, *p* = 0.002). To evaluate the relationship between the fEPSP slope and the corresponding presynaptic fiber volley amplitude, we plotted these values against each other and conducted a linear regression analysis (Fig. 3e). This analysis revealed no change in the fiber volley amplitude – fEPSP slope relationship in the juv ELE group when compared to control (*p* > 0.05) but a significant difference was present the in juv-adol ELE (*p* = 0.002) and adol ELE (*p* < 0.0001) groups. Paired-pulse facilitation experiments were also performed to interrogate presynaptic function. These demonstrated no difference between groups (Fig. 3f; *F*_(3,41)_ = 1.75, *p* = 0.171). Overall, these data suggest a significant impact of ELE on enhancing hippocampal long-term potentiation. Although this finding is in contrast to data in adult mice demonstrating lack of enhancement in CA1-LTP after chronic voluntary wheel running^9^, it may implicate a specific, 1-week period of juvenile exercise that can lead to lasting changes in hippocampal CA3-CA1 circuit function and plasticity.

## Discussion

We have successfully designed a mouse model of early-life voluntary exercise for the evaluation of hippocampus-dependent memory and synaptic plasticity during postnatal development. Our goal was to explore the possibility that physical exercise during early life can lead to enduring benefits in hippocampal function. To that end, our model was designed to target early life periods of hippocampal maturation to uncover temporally-specific cellular and molecular mechanisms uniquely engaged by early life exercise. This experimental design allowed us to test the hypothesis that juvenile exercise for one week (P21–27) is sufficient to influence hippocampal function in a lasting manner. We discovered that exercise for 4^th^-6^th^ postnatal weeks (juv-adol ELE), as well as a shorter exercise experience only during the 4^th^ postnatal week (juv ELE) enable hippocampal-dependent spatial memory formation in male mice. This was not true for mice that exercised only during the 6^th^ postnatal week (adol ELE), suggesting that the 4^th^ postnatal week of development was particularly sensitive to the exercise experience with regard to enabling long-term memory function in a lasting manner. CA1-LTP was significantly increased in mice that exercised for 3-weeks but was not further enhanced in the 1-week juvenile nor 1-week adolescent exercisers when compared to sedentary controls. An interesting finding was that properties reflecting hippocampal excitability (I/O curves) were significantly modulated in both groups of mice that exercised during the 4^th^ postnatal week; furthermore, these changes endured for at least 2 weeks in the juv ELE group. This result corresponded with the lasting effect of juv ELE on enhancing memory function in OLM, which was also demonstrated two weeks after exercise cessation. To our knowledge, this finding has not been previously tested or described in other existing models of rodent early life or adult exercise.

All mice that exercised during the juvenile period formed long-term memory of a learning event that is typically subthreshold for long-term memory formation^45^. Most notably, the effect of juv ELE on long-term memory formation persisted in both male and female mice. Our data from the juv-adol ELE group is consistent with prior work demonstrating that in adult male mice, voluntary wheel running for 3-weeks enabled memory in the same hippocampus-dependent OLM paradigm^42^. In that study, administration of the histone deacetylase (HDAC) inhibitor sodium butyrate (NaB) produced effects similar to exercise on memory and maintained memory enhancements for a longer duration than exercise. Further, both exercise and NaB treatment were associated with increased expression of BDNF as well as enriched BDNF promoter H4K8 acetylation (a marker of transcriptional activation^42,47^). These results suggest that exercise enables memory formation by modifying chromatin structure, potentially to permit transcriptional activation of genes required for exercise-induced plasticity and long-term memory formation. Previous work has alluded to this concept as a “molecular memory” of exercise^17,43,48^. In Abel and Rissman^18^, 1-week of adolescent exercise (P46–52) induced a number of hippocampal genes related to synaptic plasticity and cell signaling (including *Bdnf*, *Cbln1*, *Syn1* and *Syp*), increased global H3 acetylation, and down-regulated a number of HDAC genes^9^. Given the crucial role of histone-modifying mechanisms in memory and synaptic plasticity^49,50^, and our novel finding that juvenile exercise can lead to persistent effects on memory function, a future direction will be to assess whether there is an underlying persistence of chromatin modifications that can increase the efficiency of long-term memory formation after ELE.

Recent data suggests that adult exercise may persistently enable memory function after a brief exercise re-exposure^43^. Using the same OLM acquisition paradigm used in this study, a minimum of two weeks of voluntary exercise was required for memory enhancements to persist. Also, these memory enhancements persisted for about three days after return to sedentary activity. 2wk exercised mice given an additional 2 day “reactivating exercise” (typically insufficient for enhancing OLM performance) within a specific temporal window (seven days, but not 14 days, after return to sedentary activity) which could re-enhance memory performance in OLM^43^. This study, taken with our current findings, both highlight that timing and duration of exercise can have differing impacts on memory performance, and can reveal novel neurobiological mechanisms underlying the persistence of exercises’ effects on memory^42^.

OLM requires an intact dorsal hippocampus^46^. Enabled OLM performance in juv-adol ELE mice complements our finding of enhanced CA1-LTP in these mice. Studies focusing on the effects of exercise on LTP in hippocampal CA1 are relatively underrepresented in the literature since the finding that LTP was unchanged after chronic voluntary wheel running in adult rats^9,10^, but exercise can rescue CA1-LTP impairments in the settings of stress^13^ or sleep deprivation^14^. In this study, LTP was enhanced after theta-burst stimulation in the group of animals that underwent three weeks of exercise (juv-adol ELE), but not in those mice that underwent one-week of exercise (juv ELE and adol ELE groups, Fig. 3a). This suggests that during juvenile and adolescent periods, LTP enhancements after ELE may require long-term (greater than 1-week) exercise exposure, which has been suggested previously^9^. LTP is considered to be the cellular correlate of learning and memory^51^. Theta-burst stimulation was our chosen LTP induction protocol because this form of LTP is dependent on BDNF^52,53^, and potentially mimics circuit firing patterns occurring during learning in rodents^54^ and humans^55^. This study also presents the novel finding that hippocampal excitability was persistently augmented after juv ELE, as reflected in enhanced fEPSP slopes and fiber volley amplitudes with increasing stimulus intensity in both juv ELE and juv-adol ELE groups. Further experiments characterizing electrophysiological properties of the hippocampal network after juvenile exercise, particularly when tested well after the exercise experience has ended, would shed light on how hippocampal synaptic function may be changed in an enduring manner.

ELE has similar effects on OLM performance in females in the juv ELE (but not the juv-adol ELE) group when compared to sedentary controls. In general, voluntary aerobic physical activity in both male and female rodents during adulthood produces similar benefits in hippocampus-dependent memory tasks^56^. Although it has been well established that BDNF is one of the key mediators facilitating the effects of exercise on hippocampal function in male mice^57^, there may be sex-dependent variations in BDNF splice-variants after exercise influencing memory performance^58^. Estrogens have been shown to modulate structural plasticity within hippocampus^59^ and can enhance hippocampus-dependent memory via BDNF^60^. In the current study, adolescent females were not formally checked for stage of estrous cycling, but they could have been in varying cycle stages at the time of ELE or memory testing, thereby influencing their memory performance^61,62^.

In this study both male and female mice run similar cumulative distances regardless of exercise timing. Adult female mice typically run greater distances than males in voluntary running wheel paradigms^63^ andestrous cycling impacts running^64^. The lack of sex differences in distance ran may be due to cohort effects of phase of estrous cycling. Another interesting finding was that female stationary (sta) mice gained more weight than mice that were sedentary or exercised during the adolescent period (Fig. 1b), which could reflect skeletal muscle adaptations from climbing on the wheel. More studies are needed to characterize the physiological adaptations to ELE in male and female mice and whether they impact long-term outcomes in exercise behaviors (motivation to exercise, or the rewarding effects of exercise) and memory functions.

In summary, it is well known that exercise is a highly potent, positive modulator of cognitive function in adults. Yet exercise parameters for optimal cognitive development in typically developing children, as well as cognitively impaired children, currently do not exist. Furthermore, how early life exercise may contribute to later life cognitive functions, and the molecular mechanisms responsible for the persistent effects of ELE, is an area of research ripe for further exploration^19^. Because of the paucity of preclinical and clinical data on the subject, exercise guidelines for children are currently vague^65^. If findings in this study are relevant to humans, they implicate a lasting effect of ELE on learning and memory functions. A mechanistic understanding of ELE can be leveraged to inform exercise-based interventions for improving and preserving cognitive functions throughout the lives of children.

## Materials and Methods

### Animals

Wild-type mice were progeny of C57Bl6J dams, originally obtained from Jackson Laboratories and further bred in our animal facilities. Mice had free access to food and water and lights were maintained on a 12 h light/dark cycle. Upon weaning on postnatal day (P) 21, mice were housed in either standard bedding cages or cages equipped with a running wheel. Both male and female mice were used for all exercise and behavior experiments. Only male mice were used for electrophysiological studies. All behavioral testing was performed during the light cycle. Experiments were conducted according to US National Institutes of Health guidelines for animal care and use and were approved by the Institutional Animal Care and Use Committee of the University of California, Irvine.

### Running wheel paradigm

Upon weaning on P21, mice were pair-housed in either standard cages without a wheel or equipped with either a mobile or stationary stainless-steel running wheel (diameter 4.5 cm, 112 grams, Starr Life Sciences). Animals housed with running wheels had free access for predetermined durations: either one week during the juvenile period (P21–27; juv ELE), one week during adolescence (P35–41; adol ELE), or three consecutive weeks of access (P21–41; juv-adol ELE). An additional group of mice were housed with a stationary wheel during P21–41 (sta) to control for environmental enrichment from presence of a wheel in the cage. Each wheel was fitted with a lightweight polyurethane mesh to prevent the small limbs of weanling mice from slipping through the rungs of the wheels. Running activity was tracked via digitally monitored sensors attached to a data port and computer for real-time data acquisition (Vital View Software) via magnetic detection of the wheel revolutions. Each digitally monitored wheel tracked running distance for the cage (two mice housed per cage, thus distance traveled per cage represents the total distance ran between two mice). Revolutions were quantified by the minute daily for the duration of running and converted to distance ran per cage (km).

### Object location memory (OLM) protocol

The object location memory protocol used in this study has been adapted and modified from previous protocols^44^. On postnatal day 36, mice were brought into a testing room with reduced room brightness. Mice were handled for approximately 2 min each, for a total of 5 consecutive days prior to the OLM training session (twice a day for the first 2 days followed by once a day for the next 3 days). Habituation sessions were 5 min, twice per day for 3 days (P38-P40) and occurred within chambers containing four unique spatial cues on each wall of the chamber (horizontal lines, black X, vertical strip, and blank wall). Habituation overlapped with the last 2 days of handling. During the training phase (P41), mice were placed into the same chambers with two identical objects and exposed to either a subthreshold (3 min) or threshold (10 min) training period; a study design informed by prior work demonstrating that 3 min of training is typically insufficient for long-term memory formation^45^. For mice in exercise cages, running wheels were locked immediately after OLM training to eliminate any acute effects of exercise on memory consolidation. On testing day (P42; 24 hours after training), one of the objects was moved to a novel location inside the chamber, and mice were then allowed to explore the objects for 5 minutes. The times spent with each object was determined via blind analysis by hand-scoring, and values obtained on both training and testing days were converted into a Discrimination Index (DI), based on novel location of object during testing: [(time spent exploring novel object – time spent exploring familiar object)/(total time exploring both objects) × 100%]. There were no significant differences in memory performance between sedentary animals and those housed with a stationary wheel, so these groups were combined and represented as “no ELE” in all OLM analyses. Habituation trials were analyzed for distance traveled across sessions using ANY-maze behavioral analysis software (Stoelting Co). Mice with uneven exploration times between the two objects during the training phase (DI > 20) or showed no decrease in total distance traveled across habituation sessions were excluded from analysis.

### Electrophysiology studies

Electrophysiology experiments were performed as previously described, and the original source of these methods have been published elsewhere^66^. Only male mice were used for electrophysiological experiments. Briefly, male mice from all ELE groups were sacrificed between P42–51, and slices were collected from the rostral hippocampus. Coronal slices were sectioned (Leica VT1000 S) at 320um thickness and placed in an interface recording chamber with preheated (31  ±  1 °C) artificial cerebrospinal fluid (124 mM NaCl, 3 mM KCl, 1.25 mM KH2PO4, 1.5 mM MgSO4, 2.5 mM CaCl2, 26 mM NaHCO3, and 10 mM d-glucose). Slices were continuously perfused at a rate of 1.75–2 ml per min while the surface of the slices was exposed to warm, humidified 95% O2/5% CO2. Recordings began after at least 2 h of incubation. Field excitatory postsynaptic potentials (fEPSPs) were recorded from CA1b stratum radiatum using a single glass pipette (2–3 MΩ) filled with 2 M NaCl. Stimulation pulses (0.05 Hz) were delivered to Schaffer collateral-commissural projections using a bipolar stimulating electrode (twisted nichrome wire, 65 μm) positioned in CA1c. Current intensity was adjusted to obtain 50% of maximal fEPSP response. After a stable baseline was established, LTP was induced with a single train of five theta bursts, in which each burst (four pulses at 100 Hz) was delivered 200 ms apart (i.e., at theta frequency). The stimulation intensity was not increased during TBS. Data were collected and digitized by NAC 2.0 Neurodata Acquisition System (Theta Burst). Data in LTP figure were normalized to the last 10 min of baseline and presented as mean ± SE. Baseline measures on I/O curves, paired-pulse facilitation and LTP were analyzed using a two-way ANOVA and linear regression analysis (Graphpad Prism 8 software).

### Statistical analyses

One-way ANOVAs were used to analyze the effect of the timing of early-life exercise on OLM Acquisition DIs (Fig. 2d, g), OLM Testing DIs (Fig. 2e, h) and OLM Testing total exploration times (Fig. 2f, i) within each sex. Dunnett’s *post-hoc* multiple comparisons were used to make specific comparisons to the “no ELE” (sedentary) group when significant ANOVA results were observed. Two-way ANOVA was used to analyze mouse weight gained during the ELE period (Fig. 1b, factors of ELE group and sex). Two-way repeated measures (RM)-ANOVAs were used to analyze daily running distance (Fig. 1c–e, factors of postnatal day and sex), habituation sessions (Fig. 2b, c) and to compare OLM acquisition DI vs testing DI for each mouse for within-group comparisons. Sidak’s *post hoc* tests were used to make specific comparisons when significant interactions, or main effects of running group or OLM session were observed. Additionally, a three-way ANOVA was performed for OLM Testing DIs to determine any significant interactions between three nominal variables (OLM training duration, ELE group, and sex). For rare instances that 24 h running data for a particular day were not recorded, values were imputed based on taking the average distance ran of the prior three days, to perform statistical analyses. Unpaired Student’s t-tests were used to analyze total running distances for each ELE group (Fig. 1f–h), as well as comparing OLM testing DIs between 3-min and 10-min trained sedentary mice. Cumulative running distances are expressed as Means ± SD. Main effects and interactions for all ANOVAs are described in the text and figure legends, along with the specific number of animals of each sex used in each experiment. All analyses were two-tailed and required an α value of 0.05 for significance. Error bars in all figures represent SEM. For all experiments, values ±2 standard deviations from the group mean were considered outliers and were removed from analyses. All statistics were performed with GraphPad Prism 8 software. Total number of mice used in behavior experiments = 152.
